# Effects of ethanol on immune response in the brain: region-specific changes in aged mice

**DOI:** 10.1186/1742-2094-10-66

**Published:** 2013-05-23

**Authors:** Cynthia JM Kane, Kevin D Phelan, James C Douglas, Gail Wagoner, Jennifer Walker Johnson, Jihong Xu, Paul D Drew

**Affiliations:** 1 Department of Neurobiology and Developmental Sciences, University of Arkansas for Medical Sciences, Slot 846, Biomedical Research Building II, Room 563-2, 4301 W Markham Street, Little Rock, AR 72205, USA

**Keywords:** Aging, Brain, Chemokine, Cytokine, Ethanol, Inflammation

## Abstract

**Background:**

Alcohol abuse has dramatic effects on the health of the elderly. Recent studies indicate that ethanol increases immune activity in younger animals and that some of these proinflammatory molecules alter alcohol consumption and addiction. However, the effects of alcohol on immune activation in aged animals have not been thoroughly investigated.

**Findings:**

We compared the effects of ethanol on chemokine and cytokine expression in the hippocampus, cerebellum, and cerebral cortex of aged C57BL/6 mice. Mice were treated via gavage with 6 g/kg ethanol for 10 days and tissue was harvested 1 day post-treatment. Ethanol selectively increased mRNA levels of the chemokine (C-C motif) ligand 2/monocyte chemotactic protein-1 in the hippocampus and cerebellum, but not in the cortex of aged mice relative to control animals. In this paradigm, ethanol did not affect mRNA levels of the cytokines IL-6 or TNF-α in any of these brain regions in aged animals.

**Conclusions:**

Collectively, these data indicate a region-specific susceptibility to ethanol regulation of neuroinflammatory and addiction-related molecules in aged mice. These studies could have important implications concerning alcohol-induced neuropathology and alcohol addiction in the elderly.

## Findings

### Introduction

Alcohol abuse has significant effects on the health of the elderly population. The rates of alcoholism are high in this population. In addition, alcohol consumption in the elderly population is associated with increased motor impairment and increased withdrawal symptoms [1]. Astrocytes and microglia normally maintain homeostasis in the central nervous system (CNS) by collectively maintaining energy balance, removing toxic molecules, providing growth factors which help maintain the health of neurons, and by removing cellular debris (reviewed in [2,3]). However, when chronically activated, these glial cells can produce molecules that can be toxic to CNS cells [3]. Interestingly, a variety of studies indicate that the morphology of glia changes with aging, and these cells exhibit a more activated phenotype [4,5]. Aging glia have been demonstrated to express increased levels of inflammatory molecules including chemokines and cytokines, which are consistent with activated glial phenotypes. Further, glial activation is associated with a variety of age-related neuropathologies [4,6,7]. A series of studies indicated that alcohol increased immune activity in the CNS, which is believed to contribute to impaired neurological function and neurodegeneration associated with excess alcohol consumption. These studies indicated that alcohol increased the expression of proinflammatory cytokines and chemokines as well as cyclooxygenase-2, and nitric oxide in the CNS [8-12]. More recently, a paradigm shift regarding the role of alcohol-induced CNS inflammation occurred when it was determined that pro-inflammatory molecules including cytokines and chemokines modulate alcohol consumption, suggesting that these molecules are important mediators of alcohol addiction [13,14].

Little is known concerning the effects of ethanol on immune activity in the aged CNS. Two studies evaluated long-term ethanol exposure in aged rat cerebellum. In one study, ethanol was observed to have no effect on the density of glial fibrillary acidic protein-positive cerebellar Bergmann glial fibers or OX-42 expression by microglia relative to vehicle-treated animals [15]. In another study, chronic alcohol exposure decreased the density of Bergmann glia [16]. The reasons for the differences in experimental observations in these two studies are not clear, but likely involve differences in experimental design. More recently, the expression of the chemokine (C-C motif) ligand 2 (CCL2) was shown to be elevated in the CNS of the brains of human chronic alcoholics with a mean age of 61 to 64 years [17], which may suggest that CCL2 plays an important role in alcohol addiction.

The current study was designed to evaluate the regional effects of ethanol on immune response in the aged brain. Our study demonstrates that ethanol increases the expression of the chemokine CCL2 in a brain region-specific manner.

## Methods

### Animal treatment

Animal treatments were performed under protocols approved by the Institutional Animal Care and Use Committee at the University of Arkansas for Medical Sciences. Mice (C57BL6) were originally obtained from Jackson Laboratories (Bar Harbor, ME, USA) and were bred in-house in a Division of Laboratory Animal Medicine approved facility. Aged mice (364 to 457 days old) were treated with ethanol (15% w/v diluted from 95% v/v ethanol) or water (vehicle control) by gavage for 10 days. Ethanol was administered in two daily doses separated by 7 hours at a dose of 6 g/kg/day. Tissue was harvested 1 day after the final ethanol treatment from animals anesthetized with isoflurane.

### Blood ethanol concentrations

In a separate set of animals, blood ethanol concentrations (BECs) were evaluated using an AM1 Alcohol Analyzer as described by the manufacturer (Analox, Huntington Beach, CA, USA). In these studies, BECs were determined in mice (n = 14) 1 hour following the second dose of ethanol.

### RNA isolation and cDNA synthesis

Following anesthesia, blood was removed from the brain vasculature by briefly perfusing animals transcardially with phosphate buffered saline containing heparin. Brains were harvested, the hippocampus, cerebellum, and cerebral cortex were dissected, and the tissue immediately frozen in liquid nitrogen. The tissue was homogenized using a BBX24B Bullet Blender Blue homogenizer in a solution containing 0.5 mm RNAse-free beads (Next Advance, Averill Park, NY, USA). RNA was isolated from the homogenized tissue using a RNeasy Lipid Tissue Mini Kit (Qiagen, Valencia, CA, USA). The concentration and integrity of the resulting RNA was evaluated using an RNA 6000 Nano kit and associated Agilent 2100 bioanalyser (Agilent Technologies, Santa Clara, CA, USA). Contaminating DNA was removed from RNA samples with DNAseI (Invitrogen, Grand Island, NY, USA) and were determined to have RIN values > 8. cDNA was prepared using an iScript™ cDNA synthesis kit as described by the manufacturer (Bio-Rad, Hercules, CA, USA).

### Real-time quantitative polymerase chain reaction

The relative quantity of CCL2, IL-6, and TNF-α mRNAs was determined by real time polymerase chain reaction (rtPCR) using a CFX96 Real-time PCR Detection System (Bio-Rad). TaqMan primers were utilized in the rtPCR analysis and were synthesized by Applied Biosystems (Foster City, CA, USA). PCR reactions (20 μl) contained SsoFast™ probe supermix (Bio-Rad) and were performed in duplicate. Data were expressed as the mean ΔCt relative to β-actin. The fold expression variance of ethanol versus vehicle groups was generated by calculating the ΔΔCt. Student’s *t*-test was used to analyze variance between groups.

## Results and discussion

**Figure 1:**
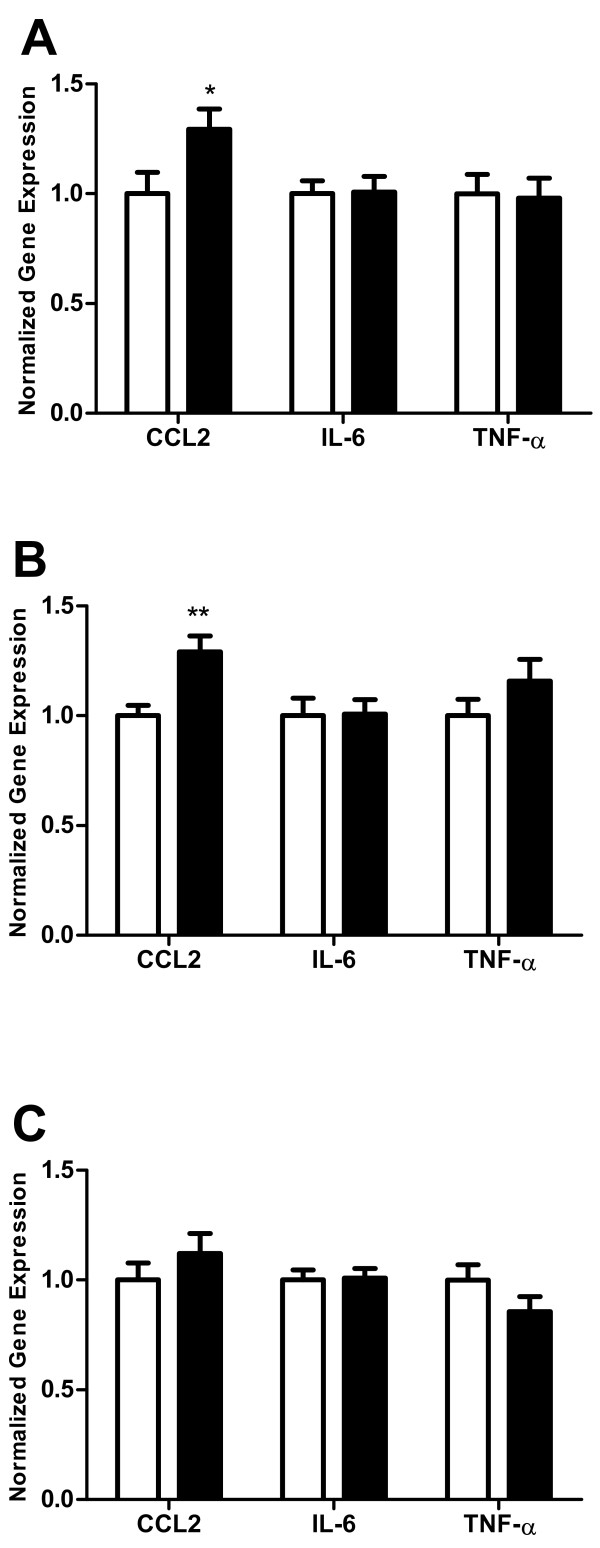
**Effect of ethanol on chemokine (C-C motif) ligand 2, interleukin-6, and tumor necrosis factor-α expression in aged mouse hippocampus, cerebellum, and cerebral cortex.** Animals were given 6.0 g/kg/day ethanol split into two doses administered 7 hours apart. Ethanol was administered for 10 days and mice were sacrificed 24 hours after the final dose of ethanol. Water was administered to vehicle mice. Hippocampus **(A)**, cerebellum **(B)**, and cerebral cortex **(C)** were isolated, RNA prepared, cDNA synthesized and mRNA levels were determined by real-time quantitative polymerase chain reaction. Results are expressed as fold changes relative to vehicle-treated mice, and all values are normalized against β-actin. Values are mean ± standard error of the mean from 9 vehicle and 12 ethanol-treated aged mice. Duplicate polymerase chain reactions were performed on each sample. *P <0.05, **P <0.01, versus vehicle-treated mice. CCL2, chemokine (C-C motif) ligand 2; IL, interleukin; TNF, tumor necrosis factor.

Ethanol is known to induce an innate immune response in the CNS, which may contribute to ethanol-induced neurodegeneration. In addition, ethanol abuse is common and has significant health consequences in the elderly population. However, the effects of ethanol on innate immune responses in aged animals have not been investigated. In the current studies, we demonstrate that ethanol increased the expression of the chemokine CCL2 in the hippocampus (Figure 1A) and cerebellum (Figure 1B), but not the cerebral cortex (Figure 1C) of aged mice. Ethanol did not increase the expression of IL-6 and TNF-α in any of these brain regions in aged mice. In a separate set of animals, BECs were determined to be 307 ± 17.43 mg/dl.

It is well established that ethanol alters innate immune activity in the developing and adult CNS (reviewed in [13,18,19]), but ethanol effects on innate immune activity are relatively unknown in the aged CNS. Our studies demonstrate for the first time that ethanol increases the expression of CCL2 in the hippocampus and cerebellum of aged mice. CCL2 expression has been demonstrated to be increased in the CNS of human alcoholics [17]. The expression of this chemokine was also increased in the whole brain of adult mice treated with ethanol [20]. Studies with CCL2 knockout mice also demonstrated that CCL2 increases alcohol consumption [21]. Collectively, our studies demonstrating increased CCL2 expression in the CNS of ethanol-treated aged mice suggest that ethanol induced neuroinflammation may contribute to neuropathology and alcohol addiction in the elderly. The current studies utilized a relatively acute model of alcohol treatment in aged animals previously naïve to alcohol exposure. Future studies are needed to evaluate the effects of chronic alcohol exposure in animals treated with alcohol during different life stages on the aged CNS. These studies may better approximate alcohol drinking behavior in elderly humans. Future studies will also need to directly compare the effects of alcohol on innate immune activity in the CNS at distinct life stages. Our unpublished studies indicate that alcohol increases the innate immune response in adult more than adolescent CNS, while the effects of alcohol on the innate immune response in the aged CNS appears to be intermediate between the adolescent and adult CNS.

It should be noted that ethanol effects on pro-inflammatory molecules were modest in the aged CNS in the current studies. For example, we did not see ethanol induction of IL-6 or TNF-α in any of the brain regions investigated. This is distinct from previous studies demonstrating induction of these cytokines in non-aged animals (reviewed in [13]). In fact, CCL2 was the only pro-inflammatory molecule that we observed to be increased by ethanol treatment of aged animals. CCL2 has been demonstrated to be expressed by neurons as well as glia. Neurons are also believed to express CCL2 receptors and respond to this chemokine. In addition, this chemokine has been demonstrated to alter synaptic plasticity in the hippocampus and is believed to suppress the toxic effects of alcohol in the CNS [22]. Collectively, this suggests that alcohol induction of CCL2 in the aged brain may represent a non-immune adaptive response to alcohol. It is possible that alcohol-induced innate immune activity in distinct regions of the hippocampus, cerebellum, and cortex were missed due to dilution effects in our analysis of these entire brain regions. There is precedence in the literature that alcohol selectively damages specific regions of the cortex, for example [23]. Additional studies are warranted to evaluate the effects of alcohol on innate immune responses in selective regions of the hippocampus, cerebellum, and cortex.

A primary purpose of the current studies was to determine if ethanol altered the expression of proinflammatory molecules in a region-specific manner in the CNS of elderly mice. To this end, we evaluated the effects of ethanol on CCL2, IL-6, and TNF-α expression in the hippocampus, cerebellum, and cerebral cortex. These brain regions are susceptible to ethanol-induced neuropathology. They also control neurocognitive and motor deficits resulting from ethanol exposure, as well as alcohol addiction. Furthermore, the structure and function of these regions is altered by ethanol exposure [24]. The hippocampus and other limbic structures play critical roles in learning, memory, and alcohol addiction [25]. Ethanol alters balance and motor coordination mediated by the cerebellum. The cerebellum and cerebral cortex mediate sedative and hypnotic effects of ethanol, which impact alcohol intoxication. Our studies demonstrate that ethanol increases CCL2 expression in the hippocampus and cerebellum. This suggests that changes in CCL2 expression may play a role in ethanol damage in key brain regions that control an array of behavioral dysfunctions associated with alcohol abuse.

The current studies demonstrated that ethanol induced CCL2 selectively in the hippocampus and cerebellum but not the cerebral cortex of aged mice. Ethanol did not increase IL-6 or TNF-α expression in any of these regions. These studies have important implications concerning ethanol-induced neuropathology and alcohol addiction in the elderly.

## Abbreviations

BEC: Blood ethanol concentration; CNS: Central nervous system; CCL2: Chemokine (C-C motif) ligand 2; IL: Interleukin; PCR: Polymerase chain reaction; rtPCR: Real-time polymerase chain reaction; TNF: Tumor necrosis factor.

## Competing interests

The authors declare that they have no competing interests.

## Authors’ contributions

JCD, GW, JWJ, and JX carried out the experiments and critically evaluated the manuscript. CJMK, KDP, and PDD designed the studies and prepared the manuscript. All authors read and approved the final manuscript.
